# Anemia Prevalence and Socioeconomic Status among Adolescent Girls in Rural Western India: A Cross-Sectional Study

**DOI:** 10.4314/ejhs.v34i1.7

**Published:** 2024-01

**Authors:** Manisha Nitin Gore, Madeline Elizabeth Drozd, Reshma Sudhir Patil

**Affiliations:** 1 Symbiosis Community Outreach Programme and Extension, Faculty of Medical and Health Sciences, Symbiosis International (Deemed University), Gram: Lavale, Tal: Mulshi, Dist: Pune, Maharashtra, India; 2 Department of Global & Community Health, George Mason University, Fairfax VA 22030, USA; 3 Department of Community Medicine, Faculty of Medical and Health Sciences, Symbiosis Medical College for Women, Symbiosis University Hospital and Research Centre, Symbiosis International (Deemed University), Lavale, Mulshi, Pune, Maharashtra 412115, India

**Keywords:** Adolescents, Anemia, India, Nutritional Status, Rural, Socioeconomic Status

## Abstract

**Background:**

Anemia poses a significant challenge among Indian adolescent girls due to their heightened vulnerability, resulting from increased micronutrient requirements, rapid physical growth, menstrual blood loss, inadequate nutrition, and socioeconomic disparities. This study sought to evaluate the prevalence of anemia, along with socioeconomic and nutritional statuses among adolescent girls attending rural public schools in Pune, India.

**Methods:**

A sample of 400 girls was selected from 22 villages through Symbiosis International University. Hemoglobin levels were assessed using the HemoCue 201 system, while standardized protocols were employed for height, weight, and BMI-for-age measurements. Socioeconomic status was determined using the Kuppuswamy scale.

**Results:**

The findings revealed an overall anemia prevalence of (42.75%), comprising severe (2.5%), moderate (21%) and mild (20.25%) cases. Additionally, a substantial proportion (74.6%) of girls were classified as underweight. Socioeconomic analysis disclosed that 64.25% of families belonged to the lower middle class, and 27% in the upper lower class. Anemia was more prevalent in young adolescent girls (10-14 years) and in the families of adolescents who had low income, were illiterate, unemployed, and belonged to the lower-middle class and upper-lower-class socio-economic status (SES) and did not have a bank account.

**Conclusion:**

Anemia was prevalent in adolescent girls and associated with low SES. This study underscores the limitations of relying solely on the distribution of iron and folic acid tablets to combat anemia. A holistic strategy is imperative, encompassing improvements in SES of families (literacy, employment and income), as well as initiatives aimed at enhancing the nutritional status of adolescent girls.

## Introduction

Anemia, a prevailing global health challenge, presents a complex interplay of physiological, socioeconomic, and environmental factors that intersect to impact the well-being of individuals and communities worldwide (1). The World Health Organization's estimate that more than 3 billion people are affected underscores the widespread reach of this issue, highlighting the pressing need to address its complex aspects. (2). While anemia's etiology is diverse, iron deficiency anemia stands out as a pivotal contributor, with its wide-ranging implications reaching across continents and populations (1).

The multiple causes of anemia collectively contribute to the vulnerability of adolescents, a group grappling with heightened micronutrient requirements during the rapid phase of physical growth (3,4). This susceptibility is magnified among young females, who contend not only with increased nutritional needs but also face additional challenges due to menstrual blood loss, inadequate dietary intake, and a matrix of socioeconomic determinants (5). The consequences of anemia among adolescents are profound and multi-faceted, encompassing compromised growth, diminished physical vitality, heightened susceptibility to infections, and increased reproductive health challenges during the transition to womanhood (6,7,8). Furthermore, cognitive development is jeopardized, and frequent illnesses coupled with physical fragility contribute to reduced school attendance, casting shadows over educational attainment (6,7,8). These repercussions extend into adulthood, undermining future labor productivity and overall societal advancement (9). Thus, anemia emerges as a concern that reverberates not only in the present but also shapes the trajectory of communities and societies (10). Recognizing the gravity of anemia's impact on community health; its mitigation has assumed critical importance on global health agendas. It has earned a significant position as a focal point in the global nutrition objectives set by the World Health Assembly for 2025 (11). Additionally, it features prominently in the Sustainable Development Goals, emphasizing its worldwide importance (12).

The global prevalence of adolescent anemia is estimated at 15% (13). A population-based study of 14,300 Indian adolescents revealed a considerably higher prevalence of 28.4%, with causes including vitamin B12 deficiency, iron deficiency, dimorphic anemia, and anemia of inflammation (14). According to the National Family Health Survey (NFHS V), females aged 10 to 19 years in India have a strikingly high prevalence of anemia (59.1%) (15), with rural areas bearing a greater burden compared to urban regions (16).

Socioeconomic status (SES) plays a significant role in the health outcomes of female adolescents in India, particularly in relation to anemia. Anemia prevalence in India is alarmingly high among this demographic. Research consistently demonstrates a strong association between lower SES and a higher risk of anemia. Low SES factors, such as poverty, limited access to healthcare, inadequate nutrition, and suboptimal living conditions, contribute to anemia's prevalence. Limited financial resources often lead to reduced food diversity, which can result in poor iron intake. Additionally, lower SES families may lack access to clean water and sanitation facilities, increasing the risk of infections that can exacerbate anemia. Furthermore, education and awareness about proper nutrition and anemia prevention tend to be lower in low SES communities. Adolescent girls from these backgrounds are less likely to receive regular health check-ups and iron supplementation. Efforts to address anemia among female adolescents in India must consider these Socioeconomic disparities (17,18,19). Association between a high prevalence of anemia among adults and factors, such as socioeconomic status and rural residence, have to be studied in detail (20). Furthermore, nutritional status significantly influences anemia prevalence in Indian female adolescents. Insufficient dietary iron intake, poor access to nutritious foods, and inadequate healthcare contribute to a high prevalence of anemia in this population (20).

In spite of the vast body of literature on anemia, a research gap remains, particularly in determining its prevalence and determinants among specific demographics. Thus, the objectives of the study encompassed the estimation of anemia prevalence and the exploration of the intricate interplay between socioeconomic status, nutritional standing, and the incidence of anemia among female students within public-funded schools in rural western Maharashtra, India.

## Methods

**Study site and population**: Symbiosis International (Deemed University) a private university in the western region of India have an outreach initiative named Symbiosis Community Outreach Programme and Extension (SCOPE), which works with an aim to positively impact the rural community, residents of 22 villages around the vicinity of the university. It strives for the achievement of sustainable, comprehensive, and integrated development of the villages with a core focus on community health. The primary objective of the study was to include every female student from government funded higher secondary schools from the 22 villages. Within this pool of villages, only five were home to government-funded higher secondary schools, which provided education from 8^th^ to 12^th^ grade.

**Sampling design and size**: Data were collected from 400 selected female students from the five higher secondary schools.

**Measures used in the study**:

- **Hemoglobin estimation** was done using Hemo Cue 201 (3). The HemoCue 201+ is validated, company calibrated and extensively employed in national surveys in India to assess the prevalence of anemia. The WHO criteria were used to define the diagnosis and severity of anemia based on the level of haemoglobin concentrations, ie. >12g/dl no anemia, 11-11.9g/dl mild, 8-10.9g/dl moderate and <8g/dl severe anemia (21).
- **Nutritional status** was assessed using the WHO's Body Mass Index, (BMI) for age values. BMI was calculated using the height and weight readings of each respondent that was captured in schools following a standardized protocol (weight in kilograms divided by height in meters squared) (22). A calibrated digital scale was used to measure the weight and a stadiometer was used to measure the height. WHO growth charts reflecting BMI for age for females aged 5 to 19 years were used as a reference for assessing the nutritional status (23). Z-score cut-points of < – 2SD, > +1SD, >+ 2SD, were used to define thinness, overweight and obese categories respectively.
- **Socioeconomic status** is “Updated Modified Kuppuswamy” scale was implemented to classify the SES status of the respondents (24). It is a composite score calculated (in the range of 3-29) on the basis of the head of the family's education, type of occupation and monthly income of the family. It categorizes populations into five groups: upper class, upper middle class, lower middle, upper lower, and lower class. The National Family Health Survey's (NFHS) guidelines were followed in collecting data on household characteristics inclusive of questions on the availability of bank accounts, health insurance, and ration cards (15).

**Data collection**: The district education officer's approval was taken before the initiation of the study in the villages. The data collection was done with the help of SCOPE's team members including clinicians, medical social workers (MSW) and staff nurses. Parents of students were interviewed by the MSWs at their residents (lists with addresses were obtained from schools) using a semi-structured interview schedule with questions on household characteristics and socioeconomic status. Measurement of height, and weight and estimation of the Hemoglobin levels of the pupils were done in school by the nurses under the supervision of the clinicians and the researchers. Data collection was done from November to February 2022.

**Data management and analysis**: The data was entered in excel and was imported into SPSS software (version 19) for analysis. Descriptive analysis was done to calculate the mean and frequencies. Chi-square and fisher exact test were used to find associations between the categorical variables; the level of significance was set at a p-value ≤ 0.05.

**Ethical considerations**: Ethics approval was taken from the Independent Ethics Committee of the Symbiosis International (Deemed University). Written informed consent was taken from the parents and written ascent was taken from students.

## Results

The mean age of participants were 14 years and half (50.8%) of the respondents were in the age group of 10-14 years and the remaining (49.2 %) were in the age group of 15-18 years. A total of 300 (90%) of the households were headed by male members, and 141 (74%) belonged to the general open caste. In context to education and occupation of the family head, 10 (2.75%) of the head of the family were illiterate, three fourth studied till middle school. A small percentage 12 (19.25%) were undergraduates. In the SES, most (64.25%) belonged to the lower middle class (III)(Table 1).

**Table 1:** Socio-demographics, socioeconomic, and characteristics of the Parents/Caregivers

Variables	n	%
Age in years (n=400)		
10-14	203	50.8
15-18	197	49.2
Household Headship (n=400)		
Male	300	90
Female	100	10
Caste of household head (n=191)		
General	141	74
Other Backward Class (OBC)	15	8
Scheduled Caste (SC)	18	9
Scheduled Tribe (ST)	17	9
Education of the head of the family (n=395)		
Illiterate (not able to read & write)	10	2.8
Schooling (1-12th Std)	383	78
Undergraduate	12	19.3
Occupation of the family head (n=395)		
Unemployed	1	0.2
Craft-related trade workers	52	13
Farming	147	36.8
Skilled Workers and Shop & Market	45	11.2
Sales Workers		
Working professionals	150	37.5
Socioeconomic status (n=389)		
Upper (I)	1	0.25
Upper Middle (II)	32	8.25
Lower Middle (III)	250	64.25
Upper Lower (IV)	106	27.25

The prevalence of anemia was 171, (42.75%) out of which 10, (5.8%), 75, (44%) and 86, (50.2%) were at severe, moderate, and mild levels respectively. Nutritional status revealed that a significant (74.6%) of children in thinness category (Table 2). No significant association was found between prevalence of anemia and nutritional status.

**Table 2:** Anemia and nutritional status

Prevalence of anemia (n=400)	n	%
Anemia	171	42.75
No anemia	229	57.25
**Grade of anemia (n=171)**		
Severe (<8gm/dl)	10	5.8
Moderate (8-10.9gm/dl)	75	44
Mild 11-11.9gm/dl	86	50.2
**Nutritional Status (n=400)**		
Normal	61	15.60
Obesity	8	2.1
Overweight	31	7.70
Thinness	300	74.60

Young age group of 10-14years were found to be anemic (*p*<0.001), Literacy levels of the heads of the families showed that 9(90%) of girls with illiterate head of the family were anemic and 12, (100%) of girls with heads of family studied till undergraduate did not had anemia. A total of 113 (74.8%) girls with heads of family into jobs had anemia, in comparison to 146 (100%) of girls with parent into farming did not had anemia (*p*<0.001). An inverse relationship was observed between family's monthly income and prevalence of anemia (*p*<0.001).

The study revealed that 106 (99%) of girls from the upper-lower socioeconomic group and 65 (26%) from the lower-middle socioeconomic group were found to be anemic, while none from the upper-middle and upper socioeconomic groups were found to be anemic(*p*<0.001). Besides, 171, (62.6%) girls' families with bank accounts, the girls had anemia. And 103(100%), with no bank accounts did not had anemia the *p*<0.001 value was found to be significant.

## Discussion

This study reflected prevalence of anemia, nutritional status, and SES of rural girls studying in the public schools of the western region of India. It highlighted the effects of SES on the prevalence of anemia among the girls.

The prevalence of anemia in the study's sample was determined to be 42.75%, demonstrating a notable contrast with the national prevalence of 59% among adolescents, as reported by NFHS-V ()(15). The review of existing literature discloses markedly elevated prevalence rates ranging from 87% to 90% in various regions within the state of Maharashtra and as high as 96% in northern states (25,26,27). The finding of this study align to some degree with the prevalence rates observed in the rural north and eastern regions of India, which stood at 43.1% and 36.4% respectively (28,29). Conversely, the eastern part of the country exhibited a comparably higher moderate-level prevalence than what was found in this study (30). In the southern regions, anemia prevalence was reported to span the spectrum of 40-80%, with a noteworthy delineation between girls attending public schools and those in private schools (31,32).

Among vulnerable demographic groups, adolescents, particularly girls in developing regions, remain prone to undernutrition, representing a multifaceted concern (15). The current study's revelation of a majority of girls falling within the thinness category raises a profound issue justifying attention. Numerous investigations have spotlighted this issue within rural and tribal pockets of India, which are marked by intricate socioeconomic challenges and a deficiency in fundamental healthcare provisions. The Comprehensive National Nutritional Survey accentuated a burden of (27.4%) for stunting and (24.4%) for thinness among female adolescents. Findings from a study in the Assamese tea garden estate unveiled stunting and thinness in (49.4%) and (50.6%) of girls, respectively, in contrast to the present study where a notably higher proportion (75%) fell within the thinness category (33). Additionally, the research highlighted that female aged 15-19 years faced elevated odds of stunted nutritional status, and adolescents from the least affluent families exhibited heightened risks of thinness (33). Notably, this investigation did not unveil any significant connection between nutritional status and anemia.

The intricate relationship between undernutrition and anemia, particularly within rural and tribal contexts, can be attributed to a gamut of factors. This includes the consumption of inadequately nutritious diets, characterized by a paucity of micronutrients and limited dietary diversity (16). Moreover, factors such as gender biased preferences in dietary allocation favoring boys, lack of awareness, dire poverty, illiteracy, and constrained healthcare accessibility all contribute to this association (16).

The Government of India has embarked on an array of public health initiatives under the umbrella of the National Health Mission, including domains such as nutrition, anemia, and other health issues with a special focus on the adolescent populace. However, it remains imperative to periodically scrutinize the operationalization and efficacy of these initiatives, particularly within the rural hinterlands of India. Our findings, which establish a pronounced link between anemia and socioeconomic status (SES), find robust reinforcement from analogous studies in India. For instance, in the southern state of Tamil Nadu, approximately 80% of anemic girls were situated within the lower socioeconomic strata, a trend mirroring our results (32). These patterns reverberate in the northern and western regions of India, in the states of Tamil Nadu, Uttar Pradesh,, and Delhi, signifying a notably elevated anemia prevalence among the less privileged Socioeconomic cohorts (34,35,36). In a study assessing the social determinants of anemia in the northwest Ethiopia, the overall prevalence of anemia among school-age children was (33.9%). Several factors were identified as associated with anemia, including mothers' illiteracy, belonging to a low-income family, experiencing stunted growth, being underweight, infection with intestinal parasites, and having a malaria infection (37).

Delving into the SES assessment of the girls' households, a salient metric was the possession of bank accounts. Intriguingly, the study's analysis revealed a higher percentage of anemia prevalence among girls' with their families having active bank accounts in comparison to those without. This phenomenon might be attributed to the governmental directives mandating the channeling of subsidies, including those for agriculture and healthcare, exclusively through bank accounts. Evidently, this policy shift has prompted a substantial upswing in the number of families establishing bank accounts, a trend corroborated by NFHS-V data. Notably, this surge is especially prominent among families from lower socioeconomic strata, as they actively sought to access the benefits of the government's subsidy programs. However, no statistically significant correlation emerged between the presence of a bank account and the SES of the families.

The intricate interplay between socioeconomic dynamics and health outcomes shows the important role of comprehensive strategies in elevating the SES of the populace. Therefore, policy interventions that holistically address socioeconomic disparities stand to yield far-reaching benefits.

The implementation of the ‘Intensified National Iron Plus Initiative’ (I-NIPI) since 2013 has been a primary endeavor to combat anemia among diverse population cohorts, ranging from children and adolescents to women in reproductive age and pregnant or lactating mothers (38). Yet, this program encounters multifaceted challenges.

In the realm of this study, certain limitations beckon attention. The study's generalizability is restricted due to its limited scope, encompassing solely five public schools from a selection of 22 villages. Additionally, the scope of the study did not facilitate further validation of the Hemoglobin estimation findings using HemoCue 201.

In conclusion, while commendable strides have been taken through initiatives like I-NIPI, the multifaceted nature of anemia demands a broader lens. The intricate interplay between socioeconomic dynamics and anemia shows the important role of comprehensive strategies in elevating the SES of the populace. Therefore, policy interventions that holistically address socioeconomic disparities stand to yield far-reaching benefits.

## Figures and Tables

**Table 3:** Associations between, demographic, Socioeconomics with anemia

Variable	Anemia		P-Value

	No	Yes	
**Age**			
10-14	32 (15.8%)	171 (84.2%)	<0.001
15-18	197 (100%)	0 (0%)	
**Education of the head of the family**			
Illiterate	1 (10%)	9 (90%)	
Schooling	218 (57%)	165 (43%)	<0.001
Undergrad	12 (100%)	0 (0%)	
**Occupation of the head of the family**			
Unemployed	0	6 (100%)	
Craft related trade workers	0	52 (100%)	
Skilled workers	45 (100%)	0	<0.001
Jobs (government, private, semi private)	38 (25.2%)	113 (74.8%)	
Farmers	146 (100%)	0	
**Income of the family (in INR)**			
<10000	1 (1%)	100 (99%)	
11000-30000	165 (69.9%)	71 (30.1%)	<0.001
310000-50000	48 (100%)	0 (0%)	
>50000	11 (100%)	0 (0%)	
**SES**			
Upper	1 (100%)	0 (0%)	
Upper Middle	32 (100%)	0 (0%)	
Lower Middle	185 (74%)	65 (26%)	<0.001
Upper Lower	1 (1%)	106 (99%)	
**Bank Account**			
Bank Account Yes	102 (37.4%)	171 (62.6%)	
Bank Account No	103 (100%)	0 (0%)	<0.001
